# Comparative quantification of chlorophyll and polyphenol levels in grapevine leaves sampled from different geographical locations

**DOI:** 10.1038/s41598-020-63407-8

**Published:** 2020-04-10

**Authors:** Elísabet Martín-Tornero, Ricardo Nuno Mendes de Jorge Páscoa, Anunciación Espinosa-Mansilla, Isabel Durán Martín-Merás, João Almeida Lopes

**Affiliations:** 1 Department of Analytical Chemistry & Research Institute on Water, Climate Change & Sustainability (IACYS), University of Extremadura, 06006 Badajoz, Spain; 2 LAQV/REQUIMTE, Laboratório de Química Aplicada, Departamento de Ciências Químicas, Faculdade de Farmácia, Universidade do Porto, Rua de Jorge Viterbo Ferreira, 228, 4050-313 Porto, Portugal; 3 Research Institute for Medicines (iMed.ULisboa), Faculdade de Farmácia, Universidade de Lisboa, Av. Prof. Gama Pinto, 1649-003 Lisbon, Portugal

**Keywords:** Leaf development, Infrared spectroscopy, Statistics

## Abstract

Near infrared spectroscopy (NIRS) and mid-infrared spectroscopy (MIRS) in combination with chemometric analysis were applied to discriminate the geographical origin of grapevine leaves belonging to the variety “Touriga Nacional” during different vegetative stages. Leaves were collected from plants of two different wine regions in Portugal (Dão and Douro) over the grapes maturation period. A sampling plan was designed in order to obtain the most variability within the vineyards taking into account variables such as: solar exposition, land inclination, altitude and soil properties, essentially. Principal component analysis (PCA) was used to extract relevant information from the spectral data and presented visible cluster trends. Results, both with NIRS and MIRS, demonstrate that it is possible to discriminate between the two geographical origins with an outstanding accuracy. Spectral patterns of grapevine leaves show significant differences during grape maturation period, with a special emphasis between the months of June and September. Additionally, the quantification of total chlorophyll and total polyphenol content from leaves spectra was attempted by both techniques. For this purpose, partial least squares (PLS) regression was employed. PLS models based on NIRS and MIRS, both demonstrate a statistically significant correlation for the total chlorophyll (R^2^_P_ = 0.92 and R^2^_P_ = 0.76, respectively). However, the PLS model for the total polyphenols, may only be considered as a screening method, because significant prediction errors, independently of resourcing on NIRS, MIRS or both techniques simultaneously, were obtained.

## Introduction

*Vitis vinifera* L. is one of the most cultivated fruit plants in the word and has a significant economic, environmental and medical impact. Grapes and wine are the major commercialized grapevine products, but the consumption of brined and fresh leaves is growing^1,2^ due to their phenolic composition and antioxidant content^3^. Moreover, grapevine leaves are a good indicator of the vigor, water stress and presence of diseases in the plant. It also reflects the grapevine cultivar or the soil where the vines are planted, being particularly useful in precision agriculture^4^. Therefore, the analysis of grapevine leaves is of paramount importance and it is essential to provide faster, more reliable and cost effective ways of diagnosing the plant status. In this sense, vibrational spectroscopy techniques, namely, mid-infrared (MIR), near-infrared (NIR) and visible (Vis) spectroscopy presents all the aforementioned properties. It is also relevant to emphasize that spectral measurements of leaves using these techniques are more robust that the measurements taken from grapes especially during the early stages of ripening.

Several reports using Vis/NIR spectroscopy applied to grapevine leaves were described in the literature. For instance, the anthocyanin content in grapevine leaves was determined in two French hybrid grapevine cultivars collected in two different months^5^. Accurate predictions were obtained despite the differences in pigments composition, leaf thickness, age and pubescence. Diseases monitoring (namely, *Plasmopara viticola*), canopy health and vigour status assessment were also successfully performed through remote sensing Vis/NIR spectroscopy system^6^. The evaluation of grapevines water status through leaves using NIR spectroscopy was already demonstrated by using portable NIR instruments. Determination coefficients around 0.90 between NIR spectra and pressure chamber measurements were obtained^7,8^. Recently, a NIR spectrophotometer mounted on a vehicle and operating without plant contact was used with the same purpose and a correlation coefficient (R^2^) of 0.88 was obtained for the estimation of the estomatal conductance^9^. Leaf relative water content has also been addressed with R^2^ between 0.66 and 0.81^10^. Infrared spectroscopy techniques have also been applied in grapevine leaves for classification purposes. Grapevine varietal and clone identification were successfully tested using the hyperspectral image of a leaf measured in reflectance mode and proper classifications around 95% were obtained in both cases^11,12^. In the same context, Gutierrez *et al*. (2015) used a portable NIR instrument for in-field grapevine varieties discrimination using the leaves spectra^13^. A total of 20 different grapevine varieties were included in this study and around 85% of correct predictions were obtained. In another work, the accuracy of Vis-NIR spectroscopy to discriminate between vineyard soils using leaves spectra was demonstrated. A comparison with the existing soil map proved that the NIR spectroscopy based estimation was very similar^14^. Moreover, the results obtained in this work confirmed that the same variety planted in different soils will grow differently, or in other words, the grapevine leaves reflect the soil where the vines are located. However, as far as we know, there are no studies regarding the discrimination of leaves of the same grapevine variety collected at different vineyards located in two geographical regions using infrared spectroscopy.

The analysis of grapevine leaves should also take into account the vegetative cycle of the plant. The vegetative cycle is a process that takes place in the vineyard each year and comprises all the morphological and biological changes. These changes are called phenological stages and their occurrence and duration is influenced by climatic factors^15^. It is known that leaf metabolites composition vary significantly over the vegetative cycle, especially during the ripening period due to environmental factors or the plant development^16^. These parameters are genetically determined, however their expression throughout the grape ripening process change with agricultural and environmental factors^17^.

In this sense, this work intends to investigate the suitability of two infrared techniques, near and mid infrared spectroscopy, for the discrimination of grapevine leaves of the same variety growing in two different geographical locations (different leaves vegetative cycles were also considered by collecting samples over different months during the ripening process), for the discrimination leaves’ vegetative cycle and also for the determination of total chlorophylls and polyphenols. and considering different vegetative cycle. The determination of these pigments was carried as their concentration can be a good indicator of leaf maturation stage and consequently an indirect indicator of grapes maturation stage. In this way, the infrared technique that prove to be the most efficient can hamper wine growers regarding the ripening stage and provide an efficient, cost-effective and multi-parametric alternative analytical tool to assist grapes ripening. This tool can also be applied into other crops.

## Material and methods

### Sample collection

Leaf sampling was carried out in two vineyards, property of SOGRAPE VINHOS SA, located in two Portuguese wine regions: *Quinta dos Carvalhais* (QC) in the Dão Wine Region (Mangualde, 40°33′28.2′′N 7°47′10.2′′W) and *Quinta da Leda* (QL) in the Douro Wine Region (Almendra, 41°01′15.1′′N 7°00′43.6′′W). All plants belong to the “Touriga Nacional” grapevine variety, a Portuguese iconic variety largely growth in the north of the country, and responsible for a large percentage of Port and Douro wine production. Eight different spots in each region were selected according to: altitude, sun orientation (solar exposition) and soil type (see Figure S1 in the supporting information).

Leaves were collected on four periods, with approximately one-month interval, from June to September (ripening period) during the 2017 campaign. Therefore, a total of 64 samples were included (8 spots at each vineyard along four periods). At each spot, a total of twenty leaves in five different plants were sampled. Once the leaves were harvested, samples were transported to the laboratory under controlled temperature conditions and stored in the freezer (−20 °C) until lyophilisation. Leaf lyophilisation was performed at −80 °C and 0.4 mbar during 3 days (Telstar, Lyoquest 85). After lyophilisation, all leaves from each sampling spot were mixed and milled. Then, the samples were stored at room temperature in the dark.

### Chemical analysis

#### Total phenolic compounds

Polyphenols were extracted from approximately 0.25 g of milled samples with 10 mL of methanol:water (80:20) in an ultrasound bath during 30 minutes. The obtained extract was centrifuged at 3000 rpm for 10 min and the supernatant was used to determine total phenolic compounds.

Total phenolic compounds were determined colorimetrically using a Cary 50 UV-VIS spectrophotometer (Agilent Technologies) following the method described by Singleton and Rossi (1965)^18^. Aliquots of 1 mL of the extract diluted 1:33 (v/v) with ultrapure water, or gallic acid standard was added into a 10 mL borosilicate tube, followed by additions of 5 mL Folin-Ciocalteu reagent (1:10 v/v with water) and 4 mL of 75 g L^−1^ sodium carbonate solution. After mixing, the samples were incubated for 1 h at room temperature and the absorbance of the mixture was measured at 760 nm using the respective mixture with only ultrapure water as blank. External standard methodology was used. Total phenolic compounds content were expressed as mg of gallic acid per g of lyophilized leaves.

#### Chlorophylls a and b

Pigments, chlorophylls and carotenoids, were extracted from leaves with methanol. Accurately weighted 0.1 g of lyophilized leaves was mixed with 10 mL of methanol in a centrifuge tube. After mixing with a vortex, all tubes were placed in an ultrasound bath for 15 minutes and centrifuged at 3000 rpm for 10 minutes. Then 300 μL of supernatant was diluted to 3 mL with methanol. The absorbance of this solution was measured at 470, 652 and 665 nm and the concentrations of chlorophylls and carotenoids pigments were determined by using equations according to^19,20^.

### Spectral acquisition

NIR spectra of lyophilised grapevine leaves powder were collected in diffuse reflectance mode on a Fourier-transform near infrared spectrometer (FTLA 2000, ABB, Quebec, Canada) equipped with an indium-gallium-arsenide (InGaAs) detector and controlled by Bomen-Grams software (version 7, ABB, Quebec, Canada). All samples were transferred to borosilicate flasks prior to spectral acquisition. Each spectrum resulted from an average of 64 scans with a resolution of 8 cm^−1^ within the spectral range of 10000 to 4000 cm^−1^. Three spectra of each sample were collected and the average was considered for further analysis. Teflon reference material was used as background.

MIR spectra of the lyophilised grapevine leaves powder were collected in diffuse reflectance mode on a Fourier-transform spectrophotometer (Spectrum BX FTIR, PerkinElmer, Waltham, USA) equipped with a DTGS detector and PIKE Technologies Gladi ATR accessory. Three portions of each sample were transferred to the ATR crystal and compressed with a pressure of 150 N cm^−2^ for spectral acquisition. The resultant spectrum was obtained from an average of 32 scans with a resolution of 4 cm^−1^ within the spectral range of 4000 to 600 cm^−1^. The ATR crystal was cleaned, dried and a background (empty cell/air) was performed between each grapevine leave sample measurement. All spectral measurements were performed in triplicate and the average was considered for further analysis.

### Data analysis

All spectra (NIR and MIR) were pre-processed in order to remove variations and artifacts that do not represent the actual differences between samples. In this work, both NIR and MIR spectra benefit from baseline correction. Therefore, a Savitzky-Golay filter with a filter size of 15 points, 2nd order polynomial, and first or second derivative was applied followed by standard normal variate (SNV). These methods were thoroughly used in the literature for similar spectral data processing^14^. After this, all the spectra were mean centered for further analysis.

Principal component analysis (PCA) was carried out prior to classification and quantification approaches, with the objective of detecting outliers and eventually evaluate possible clusters formations (exploratory data analysis)^21^. Leaves classification was carried out by partial least squares discriminant analysis (PLS-DA). Several PLS-DA models were built with different classification purposes, namely for geographical region discrimination and vegetative stage discrimination. Samples were divided in two sets. The first data set (training set) was composed by leaves collected from 6 spots of each vineyard. The training set was used to perform the calibration and the cross validation of the models. The other dataset (test set) was composed of leaves collected from the remaining 2 spots and it was only used to test the robustness and accuracy of the developed PLS-DA models. This data division was only performed for the PLS-DA models. With the aim to select the most significant spectral regions for the discrimination of the geographical region and vegetative stage, NIR and MIR spectra were divided in five different regions. For NIR, the spectral regions were: 9960 − 7317 cm^−1^ (R1), 7314 − 6507 cm^−1^ (R2), 6503 − 5350 cm^−1^ (R3), 5346 − 4964 cm^−1^ (R4), 4961 − 4035 cm^−1^ (R5). The spectral regions established for MIR were: 3982 − 2702 cm^−1^ (R1), 2700 − 1802 cm^−1^ (R2), 1800 − 1182 cm^−1^ (R3), 1180 − 862 cm^−1^ (R4) and 860 − 620 cm^−1^(R5). Therefore, the optimization of the PLS-DA models involved selecting the optimal number of latent variables (LV’s) according to the lowest cross validation error using venetian blinds technique. The (combination) of best spectral regions were evaluated by performing all possible combinations using only the training set and the cross-validation strategy. After this optimization step, the validation set was projected into the training set and PLS-DA predictions were compiled under the format of a confusion matrix (presenting only for the validation set results). The percentage of correct predictions was obtained through the diagonal elements sum of the confusion matrices.

Moreover the partial least squares (PLS) regression was used to establish prediction models for total chlorophylls and polyphenols based on spectral data (NIR and MIR techniques). The entire NIR and MIR spectra were considered and the samples were randomly divided into two sets: 70% of the samples for calibration (45 samples) and the rest (30%) for validation (19 samples). This was done ensuring that the samples included in the validation set presented parameter values within the ones found in the calibration set (a kennard-stone algorithm was employed). Calibration optimization was achieved by estimating the best number of latent variables according to the leave-one-sample-out cross validation procedure and testing different pre-processing techniques (namely, SNV, Savitzky-Golay filter with different filter widths, polynomial orders and derivatives) individually and in combination using only the calibration set. After this optimization, the prediction set was projected and the PLS models evaluated using the following parameters: root mean square error of calibration (RMSEC), root mean square error of cross validation (RMSECV), root mean square error of predictions (RMSEP), coefficients of determination for cross validation (R^2^cv) and prediction (R^2^p), range error ratio (RER), residual predictive deviation (RPD), limit-of-detection (LOD) and limit-of-quantification. More details can be found at^22^.

Data analysis was performed using Matlab R2016b version 9.1 (The Mathworks Inc. Natick, MA, USA) with the PLS_Toolbox 8.2.1 (Eigenvector Research Inc. Wenatchee, WA, USA).

## Results and Discussion

**Figure 1:**
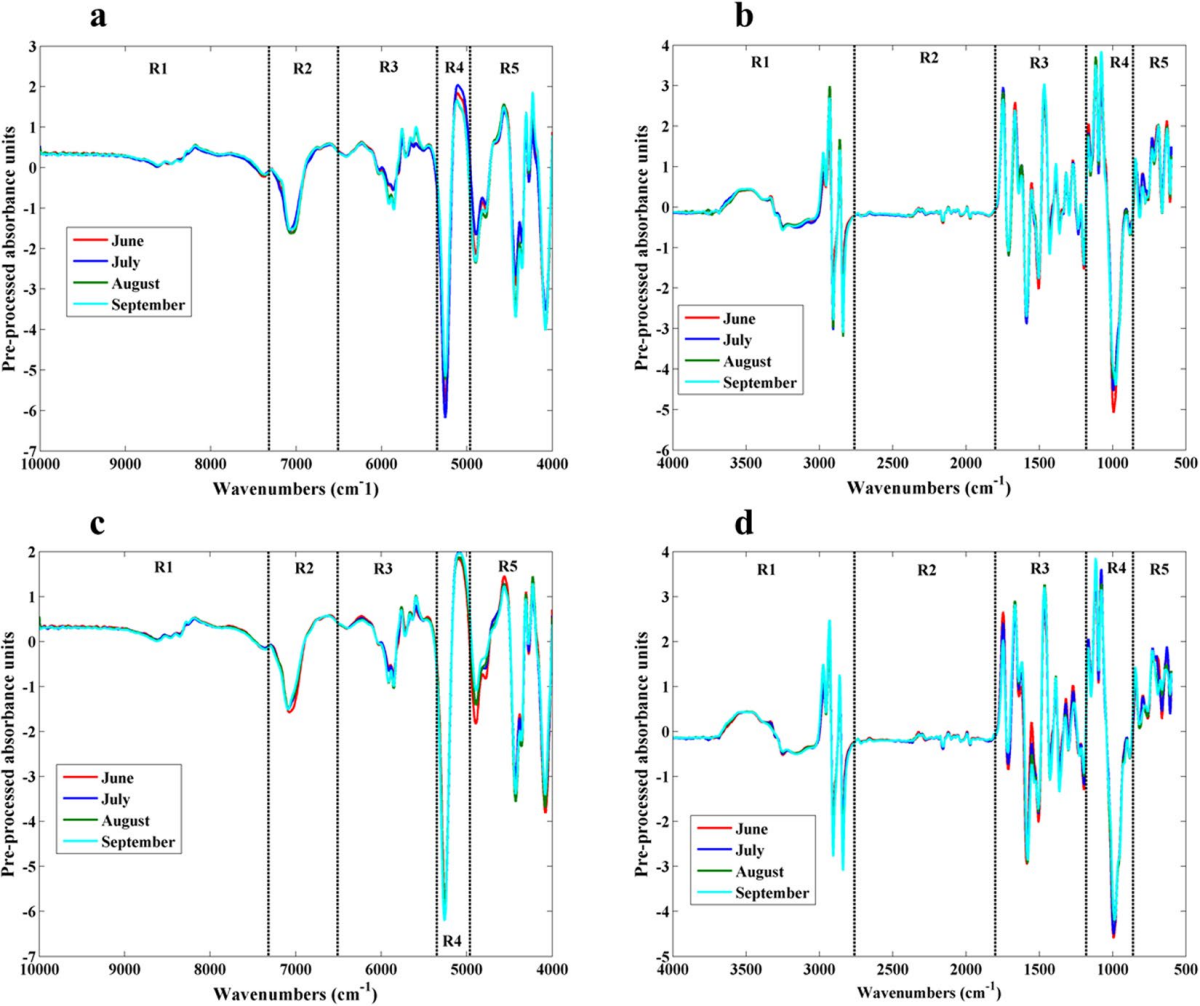
Average of pre-processed NIR (**a,c**) and MIR (**b,d**) spectra (Savitzky-Golay using a window size of 15, 2nd polynomial order and first derivative followed by SNV) of leaves samples collected in the different sampling dates and in two different geographical regions, Quinta dos Carvalhais (**a,b**) and Quinta da Leda (**c,d**).

Grapevine leaf composition is influenced by soil properties and the vegetative stage. Thus, the composition of leaves could be allow the discrimination between grapevines of different geographical zones. With the aim to obtain information about the composition of grapevine leaves, NIR and MIR spectra from the lyophilized samples were obtained. Figure 1 shows the average of pre-processed NIR and MIR spectra of leaves collected from two vineyards during the vegetative cycle, comprised between June and September.

The spectra of grapevine leaves are complex, as can be observed, showing many bands which reflect the complex composition of the grapevine leaves. Grapevine leaves vibrational spectra showed differences according to the geographical origin (QC and QL) and vegetative stage. Regarding leaves NIR spectra, it is possible to obtain information concerning several chemical compounds commonly present. The chemical compounds commonly present in leaves such as carbohydrates (e.g., cellulose, starch), lipids, proteins, water and phenolic compounds (e.g. lignin), have already specific bands assignment in NIR spectra^23^. Moreover, other chemical compounds that contain or influence the following chemical bonds, C-H, N-H, O-H and S-H, can also be detected. In relation to MIR spectra, the identification of the bands is less complex than in NIR spectra but the chemical compounds that can be detected are similar to the ones above mentioned.

### Geographical analysis

**Figure 2:**
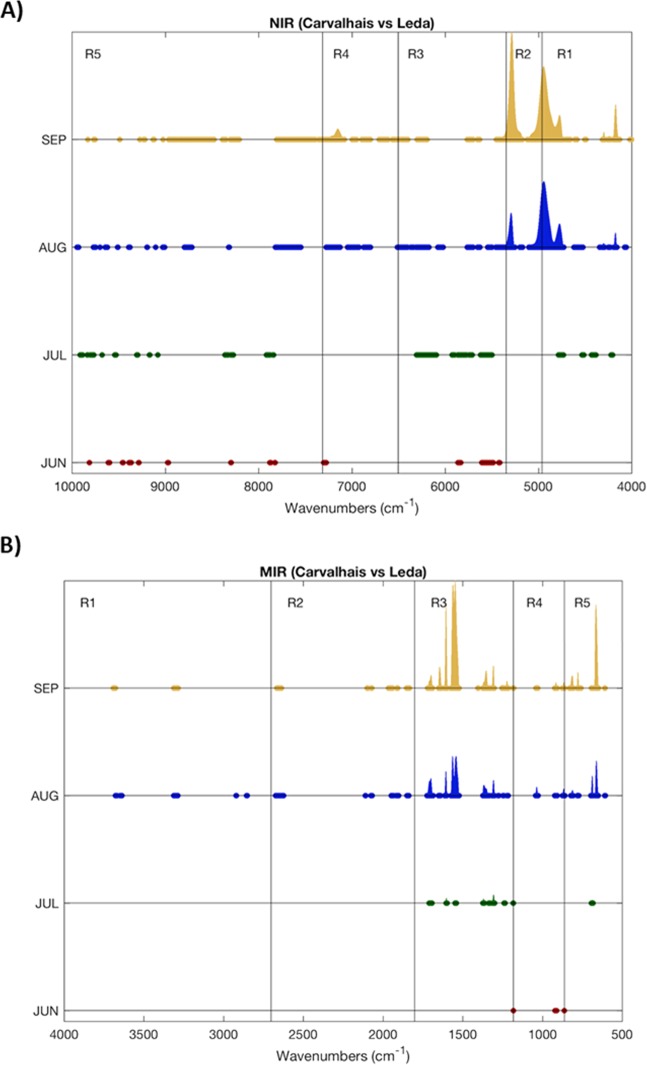
Identification of the significant spectral differences regions found between both geographical origins. Results considering NIR (**A**) and MIR (**B**) spectra segregated according to the month are shown.

Both NIR and MIR spectra acquired from the two different vineyards were compared in order to highlight significant differences and the eventual impact of the two terroirs on the characteristics of the leaves. The adopted procedure was based on the initial pre-processing of all spectra according to the selected method (section 2.4 Data Analysis) and then by calculating the average and standard deviation of the spectra for each vineyard. Wavenumbers, where a significant difference was found, were marked and the difference between the averages was performed. This procedure reveals regions in the spectra where there is a significant difference (two standard deviations were considered), in this case considering a 0.05 significance. This procedure was performed considering the months of June, July, August and September separately. For a better visualization of the differences, for each technique the average differences were normalized (divided by the highest computed difference) (Fig. 2).

The obtained results using NIR and MIR techniques were in agreement. First, it is clear a very high degree of similarity between spectra of both geographical origins for the months of June and July. Conversely more pronounced differences were observed in August and September. Indeed, considering the last two periods, a very important number of spectral windows revealed significant differences (>5200 cm^−1^ for NIR and >2100 cm^−1^ for MIR spectroscopy). It should also be noted that using NIR spectroscopy it was possible to capture differences almost in the entire spectrum for the month of September, although not so remarkable as those found above 5200 cm^−1^.

**Figure 3:**
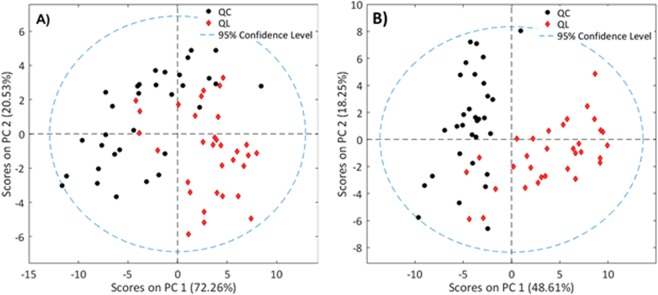
PCA score plots of the two first components obtained for NIR (**A**) and MIR (**B**) spectra for both geographical origins discrimination considering the entire spectral range and the complete sample data set.

A PCA of both NIR and MIR spectra was performed to examine the composition changes on the leaves of the same variety grown in the two different geographical regions. PCA models for each set of spectra were built in order to detect any grouping of samples. This exploratory analysis was performed applying first-order derivative, SNV as data pre-processing in the entire spectral regions. Mean-centering was always used as scaling procedure previous PCA analysis. The results show that the two first components explain more than 92% of the spectral variance in NIRs, and more than 66% in MIRs. The PCA score plots of the first two principal components (PC1 and PC2) for each model (NIRs and MIRs) are shown in Fig. 3.

As can be seen in Fig. 3, both score plots show some tendency to cluster samples in two groups, highlighting and confirming chemical differences between leaves collected from different geographical regions. For the NIRS model, most of the samples from QL are placed in the fourth quadrant and the samples from QC are distributed between the others three quadrants. However, as can be seen in Fig. 3A, some samples of QL are placed between the samples of QC. When these samples were identified, all of them correspond to samples collected in June and July.

For the MIRs model, the groups are better defined. Most QC samples are placed in the negative side of PC1 in opposition to samples from QL. Some overlapping can also be observed in the negative side of the PC2. These samples correspond to the June month, suggesting that lower differences are found between the samples collected in June. In order to confirm this, two additional PCA models were built for each spectral data set, considering only June and September. The score plots of the two first principal components for each model can be seen in Figure S2 of the supporting information. These plots confirm that better discrimination between leaves collected in the two vineyards is found for samples harvested in September. The promising results of the exploratory data analysis suggest the use of a supervised classification method for the discrimination of the geographic origin.

The supervised classification method selected was PLS-DA. In order to select the spectral region that achieve better discrimination between the two classes, a PLS-DA model was developed for each separated spectral region and for the entire spectra using in all the cases Savitzky-Golay with 15 points filter width, 2^nd^ polynomial order and first–derivative followed by SNV as pre-processing. The number of LVs was tested until a maximum of 10. Table 1 shows the % of correct predictions considering different number of LVs included in the PLS-DA model performed for each separated region and for the entire spectra, both with NIRS and MIRS. In this case, separated spectral windows were tested. Results are described for the prediction set.

**Table 1:** Percentage of correct predictions for geographical origin discrimination obtained through PLS-DA considering individual spectral regions and the entire spectra for different number of latent variables (LV). All the samples were used (n = 64).

LV	Entire spectra	R1	R2	R3	R4	R5
	Near infrared spectroscopy					
1	81	81	81	94	81	81
2	94	100	81	81	94	94
3	94	100	94	94	94	94
4	100	100	100	94	100	100
5	100	100	100	100	100	100
	**Mid infrared spectroscopy**					
1	94	56	81	100	100	94
2	94	88	88	100	100	88
3	88	88	94	94	94	88
4	94	94	88	94	94	88
5	94	100	88	94	94	88

The percentage of correct predictions obtained for each spectral region with both techniques indicates whose spectral regions could be more important. As can be seen in Table 1, NIR spectra show a slightly better performance than MIR spectra for geographical origin discrimination. Moreover, all the spectral regions of NIR and MIR spectra showed a percentage of correct predictions around 90–100% with a low number of LVs. In this context, these results reinforce the idea that “terroir” has a high impact over the grapes and leaves of grapevines as leaves from different “terroirs” present a different chemical composition.

**Figure 4:**
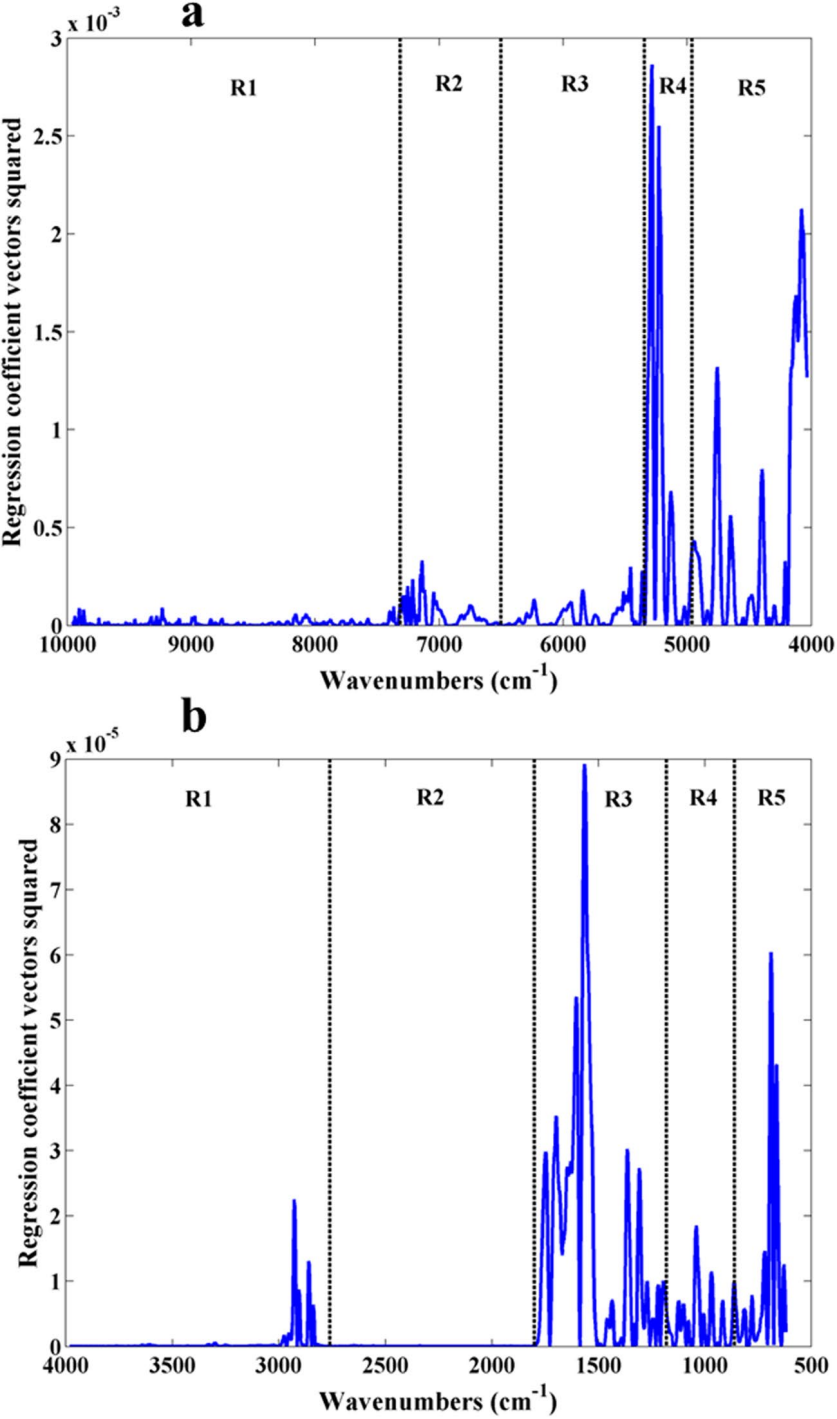
Squared PLS-DA regression coefficient vectors model for geographical discrimination considering a 4 LV NIR model (A) spectra, and a 1 LV MIR model (B). Note that for this PLS-DA model the regression coefficients for both classes are symmetric, and therefore the square is the same.

The analysis of the regression coefficient vectors of the PSL-DA models will allow a better visualization of the most important spectral regions. In Fig. 4A, the regression coefficient vectors considering the entire NIR spectra and 4 LV were plotted, and the respective confusion matrix was shown in Table 2. The same was done considering the entire MIR spectra and 1 LV (Fig. 4B and Table 2).

**Table 2:** Confusion matrix obtained for a PLS-DA model for vineyards discrimination. All the samples were used (n = 64).

Vineyard (predicted)	Vineyard (real)				
	NIR (LV = 4)		MIR (LV = 1)		Total
	Quinta dos Carvalhais	Quinta da Leda	Quinta dos Carvalhais	Quinta da Leda	
Quinta dos Carvalhais	50	0	50	0	50
Quinta da Leda	0	50	6.3	43.7	50
Total	50	50	56.3	43.7	100

Values are expressed in percentage considering total samples.

The more important wavenumbers for geographical origin discrimination obtained by the PLS-DA model using NIR spectra were located within the region 5,500 to 4,000 cm^−1^. These wavenumbers belong to the NIR combination band region where N-H plus C-H, C-H plus C-H and C-H plus C-C bonds vibrations are located and therefore can be mainly associated with carbohydrates and proteins^24,25^. It makes sense that the “*terroir effect”* has a significant impact over several chemical compounds commonly present in grapevine leaves, namely carbohydrates and proteins. Regarding the regression coefficient vectors squared obtained by the PLS-DA model using MIR spectra for geographical origin discrimination, the wavenumbers with the highest contribution were located around 2,900 cm^−1^, within 1,800 to 1300 cm^−1^ and around 700 cm^−1^. The wavenumbers around 2,900 cm^−1^ can be attributed to C-H bonds of carbohydrates^23^ while the wavenumbers within 1,800 to 1300 cm^−1^ can be related with several molecules, such as amino acids and/or proteins due to the amide group at 1655 and 1565 cm^−1^^23,26^, chlorophyll due to the C = O band around 1700 cm^−1^^26^ and cellulose due to the C = O, O-H and CH_2_ bands within 1750 to 1435 cm^−1^^23^. The wavenumbers around 700 cm^−1^ can be related with proteins namely glycine due to CH_2_ and NH_2_ bonds and lipids due to CH_2_ bonds^26^.

The confusion matrix obtained considering the entire NIR spectra revealed that all leaf samples were correctly classified. When the entire MIR spectra was used, all leaf samples from Quinta dos Carvalhais were correctly classified while around 6% of the samples belonging to Quinta da Leda were misclassified as belonging to Quinta dos Carvalhais.

### Leaf vegetative stage analysis

**Figure 5:**
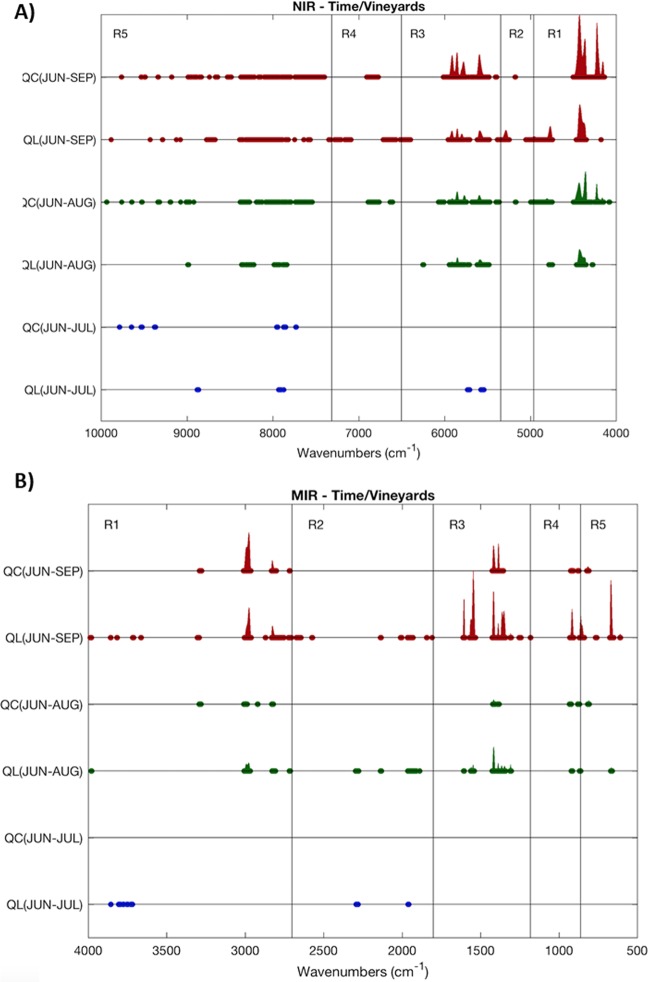
Identification of the spectral regions where statistically significant differences were found between NIR (A) and MIR (B) spectra considering different time periods. The comparison is always made considering the June month as the basis.

A similar analysis was performed along the leaf vegetative stage. It was important to evaluate how spectral markers differences evolve over time and how this evolution can be distinct considering different geographical origins. The same procedure was followed, but now considering differences between the spectra collected in June in relation with the remaining three months. In Fig. 5, the processed spectra obtained by both techniques along leaf vegetative stage and in the two geographical regions are shown.

The analysis of the leaf vegetative stage from the point of view of the spectral data reveals that only marginal differences are observed between data collected in June and July. However, as the time span increases significant differences emerge, and this observation was valid for both NIR and MIR spectroscopy. Differences in multiple spectrum locations appear to increase as the time span increases. This was observed in both vineyards. Nevertheless, and taking into account the magnitude of the differences, it can be mentioned that the spectral differences between the same spectral window for QL and QC are similar but not exactly the same, which in some degree validates the observation made before, when the two geographical origins were compared and considerable differences were observed. This finding highlights that the same grapevine variety planted in different geographical regions presents different chemical profiles in the leaves. A PCA model was carried out to explore the data and to detect grouping of samples collected in the four sampling dates at each geographical region. The exploratory analysis was performed under the same pre-processing conditions as for previous PCA models. The score plots obtained for the first two principal components for both techniques (NIRS and MIRS) and geographical regions (Figure S3 supporting information) allowed to distinguish between two groups, samples of June and July are located on one side of the PC1, while the samples of August and September are located on the other side, regardless of the geographical region and the technique used.

Due to the observed differences, a PLS-DA model was built for each geographical region. To find the best spectral region for this discrimination and the optimal number of LVs, the same procedure abovementioned was adopted. It was possible to conclude that for both NIR and MIR spectra there were no significant differences between the percentage of correct predictions for the leaf vegetative stage discrimination obtained for each separated spectral region and the entire spectra.

In this context, the confusion matrix considering the entire NIR spectra of leaves from Quinta dos Carvalhais and Quinta da Leda with 5 LV and 4 LV, respectively is shown in Table 3. With MIR spectra, the entire spectra were also used and 2 LV for Quinta dos Carvalhais and 3 LV for Quinta da Leda were considered.

**Table 3:** Confusion matrix obtained through PLS-DA model for leaf vegetative stage discrimination. Values are expressed in %. All the samples for each vineyard were used (n = 32).

Leaf vegetative stage (predicted)	Leaf vegetative stage (real)									
	Quinta dos Carvalhais					Quinta da Leda				
	Near infrared spectroscopy									
	June	July	August	September	Total	June	July	August	September	Total
June	25	0	0	0	25	12.5	12.5	0	0	25
July	0	25	0	0	25	0	12.5	12.5	0	25
August	0	0	12.5	12.5	25	0	0	25	0	25
September	0	0	0	25	25	0	0	0	25	25
Total	25	25	12.5	37.5	100	12.5	25	37.5	25	100
	**Mid infrared spectroscopy**									
	**June**	**July**	**August**	**September**	**Total**	**June**	**July**	**August**	**September**	**Total**
June	25	0	0	0	25	25	0	0	0	25
July	0	25	0	0	25	0	12.5	12.5	0	25
August	0	0	25	0	25	0	0	12.5	12.5	25
September	0	0	12.5	12.5	25	0	0	12.5	12.5	25
Total	25	25	37.5	12.5	100	25	12.5	37.5	25	100

The percentages of correct predictions obtained for Quinta dos Carvalhais and Quinta da Leda were 87.5% and 62.5%, respectively, considering both techniques.

Both techniques allowed a correct classification between the samples collected in different months at both geographical regions, taking into account that the different compounds evolve continuously throughout the leaf vegetative stage. Therefore, as can be seen by the confusions matrices, the misclassified samples were only obtained between two consecutive months which can be easily explained by the small chemical composition difference between these leaves. However, no samples from June were classified in September and vice versa.

### Correlation between infrared spectra and physicochemical analysis

Chlorophylls are among the most abundant pigments in photosynthetic plants. Their content provides valuable information about the physiological status of the plant and the relation of all photosynthetic pigments are important indicators of leaf senescence^27^. From all chlorophylls, chlorophyll *a* and *b* are the more predominant and for this reason were the ones analyzed in this study. On the other hand, the total polyphenol content was analyzed because phenolic compounds are known to play a key role in the final quality of grapes and wine.

PLS regression was selected to establish quantitative models between the chemical analysis of total chlorophylls (refers to the sum of chlorophyll a and b) and total polyphenols (expressed as gallic acid) obtained through reference procedures and the entire spectral data of NIR and MIR techniques as aforementioned. The PLS models were optimized using the calibration set (section 2.4) and four different PLS models were built (two using the experimental data and NIR spectra and the other two using the experimental data and MIR spectra). The best pre-processing technique obtained for the determination of total chlorophylls and polyphenols considering NIR spectra was Savitzky-Golay with 15 points filter width, second polynomial order and second derivative, followed by SNV. For MIR spectra, the application of SNV generated the best results for both total chlorophylls and polyphenols determinations.

**Figure 6:**
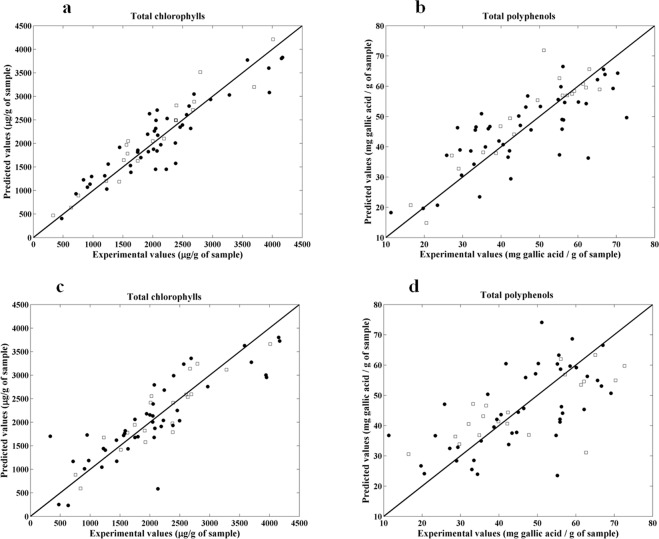
Experimental values versus PLS cross-validation predictions (●) and independent test set predictions (□) for total chlorophyll (**a**) and polyphenols (**b**) using NIR spectra and for total chlorophyll (**c**) and polyphenols (**d**) using NIR spectra and MIR spectra.

A graphical representation of the experimental versus predicted total chlorophyll and total polyphenol content is presented in Fig. 6.

The PLS results are shown in Table 4. The best PLS models were obtained for total chlorophyll determination by both techniques, and NIR revealed to be more accurate. The NIR PLS model results suggest that it is possible to determine the amount of total chlorophyll (R^2^_P_ of 0.92, RER of 13.0 and RPD of 3.41) with a relatively high accuracy (RER and RPD above 10 and 2.5, respectively), while for total polyphenols (R^2^_P_ of 0.76, RER of 7.50 and RPD of 1.90) it can be used only as a screening method. Regarding MIR data, the PLS model results reveal that this technology is also accurate for the determination of total chlorophyll (R^2^_P_ of 0.84, RER of 10.2 and RPD close to 2.5) while for total polyphenols the result follows the trend already observed for NIR spectroscopy (R^2^_P_ of 0.51, RER of 5.15 and RPD of 1.46). The other figures of merit calculated reinforce that NIR spectroscopy is more accurate.

**Table 4:** PLS calibration models’ results for total chlorophyll and polyphenols using NIR and MIR spectroscopy. All the samples were used (n = 64).

	Total chlorophylls (µg g^−1^ of sample)		Total polyphenols (µg g^−1^ of sample)	
	NIR	MIR	NIR	MIR
LV	5	5	4	8
RMSEC	204	375	6.33	6.72
RMSECV	355	504	9.51	11.8
RMSEP	283	318	7.87	10.9
R^2^_CV_	0.84	0.72	0.61	0.40
R^2^_P_	0.92	0.84	0.76	0.51
RER	13.0	10.2	7.5	5.2
RPD	3.41	2.48	1.90	1.46
Slope	Y = 0.95x + 134.3	Y = 0.86x + 253.4	Y = 0.87x + 5.57	Y = 0.56 + 19.9
Bias	−2110	2635	18.7	12.7
LOD	849	954	23.6	32.7
LOQ	2830	3180	78.7	109

**Figure 7:**
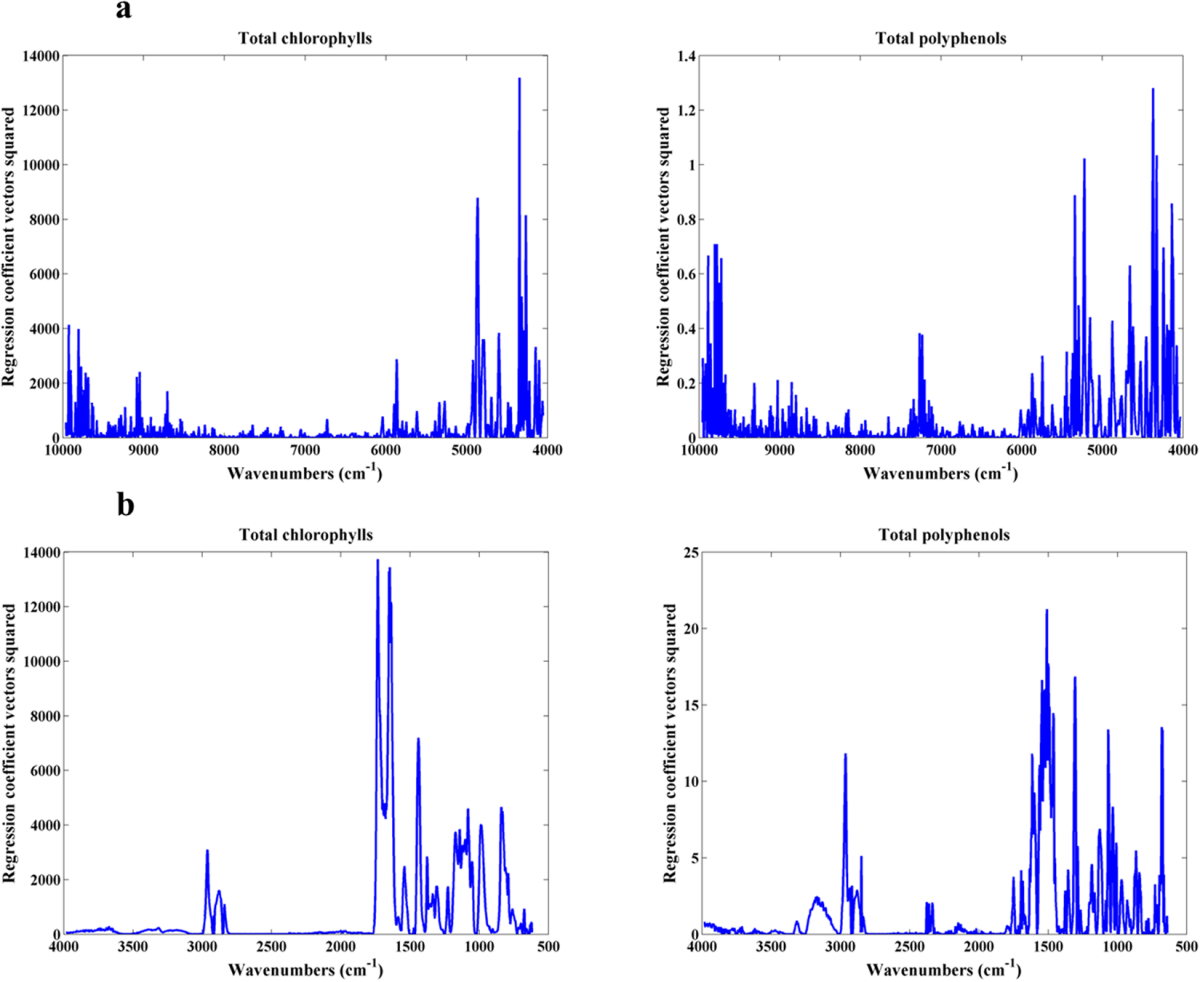
Squared regression coefficient vectors of total chlorophyll and polyphenols PLS models built using the entire NIR spectra (**A**) and the entire MIR spectra (**B**).

Besides presenting the PLS model’s results it is also important to understand which spectral regions have a higher contribution to the respective models. In this context, the regression coefficient vectors of all PLS models were plotted in Fig. 7.

For NIR spectra and considering the total chlorophyll, the most important wavenumbers were located within the region between 5,000 and 4,000 cm^−1^ while for total polyphenols the most important wavenumbers were located within 6,000 to 4,000 cm^−1^. The wavenumbers associated with the chlorophyll content are commonly located at the spectral regions closer to the visible region^24^. In this case, the wavenumbers within 5,000 and 4,000 cm^−1^ are usually associated with carbohydrates and proteins content present in the leaves^24,25^. In fact, this spectral region belongs to the NIR combination band region where N-H plus C-H, C-H plus C-H and C-H plus C-C bonds vibrations are located. However, this could make sense because the chlorophyll molecule has all these bonds and another work suggested the possibility of chlorophyll being responsible for absorptions within the spectral region of 4,800 to 4,300 cm^−1^^28^. Regarding the total polyphenols content, the wavenumbers located within 6,000 to 4,000 cm^−1^ include the NIR combination band region abovementioned and part of the first overtone region, including the S-H bonds. This entire region can be associated to aromatic compounds and phenols^29^.

Considering MIR spectra and total chlorophyll PLS model the most important wavenumbers were located within 1,770 to 800 cm^−1^ while for total polyphenols PLS model the most important wavenumbers were located within 1,650 to 600 cm^−1^. For total chlorophyll, the highest peaks were located around 1,700 cm^−1^ which are in agreement with C = O bands of chlorophyll molecule^26^. Regarding total polyphenols, the wavenumbers within 1600 and 1100 cm^−1^ can reflect the presence of aromatic compounds, flavonoids and phenols^23,30^. Thus, this reinforces why the more important wavenumbers for total polyphenols were found within 1,650 and 600 cm^−1^.

## Conclusions

High-throughput analysis resourcing on vibrational spectroscopy methods such as near or mid-infrared spectroscopy for the monitoring of vineyards has been proposed essentially aiming at following grapes ripening processes and for harvest time tracking. These procedures made on grapes, both *in-situ* or at the lab, present sampling difficulties given the geometry, high water content and variability of grapes chemistry within bunches. This work evaluated the feasibility of tracking the plant evolution by using the same spectral methodologies but applied on dried (lyophilized) leaves, thus increasing signal-to-noise and eliminating the water content disturbance. Results obtained from leaves of the same grape variety collected from two geographically different wine regions over four months (June to September) show that it is indeed possible to track the ripening stage. Signals collected with NIR and MIR both allow to differentiate the geographical origin of grapevine leaves with an accuracy above 95% independently of the ripening month. Moreover, differences known in grape chemical profiles from these wine regions caused by a distinct terroir can also be detected from grapevine leaves. The evolution of leaves chemical composition during the June-September period could also be tracked by NIR/MIR spectroscopy. Markedly differences are observed when June leaves spectra are compared with August and September leaves spectra, indicating a major chemical profile transition around end of July. Besides using the spectra to track the vinegrape leaves evolution during the ripening period, the quantification of total polyphenols and chlorophylls was also successful. For quantification purposes, NIR spectroscopy revealed a better option with and higher accuracy when compared to MIR spectroscopy (quantification errors approximately 20 to 30% lower). The application of these methods to the determination of chlorophylls was also superior compared to the total polyphenols with almost twice high range-error-ratios. Quantification accuracy for chlorophylls and polyphenols yield 283 and 7.9 µg/g, respectively. This work demonstrated that it is possible to track vinegrape chemical evolution over the ripening period by monitoring the leaves (dried leaves), leaving an open possibility to use this methodology for monitoring the grapes ripening process. As already demonstrated in the literature, the *in-situ* spectra analysis of leaves is much more robust when compared with grapes, thus requiring substantially fewer spectral analyses, and allowing the monitoring of a increased areas in the same time period.

## Supplementary information

Supplementary information
