# Supplementary material for: Comparative quantification of chlorophyll and polyphenol levels in grapevine leaves sampled from different geographical locations
Source: Sci Rep. 2020 Apr 10;10:6246. doi: 10.1038/s41598-020-63407-8 (PMC7148322; doi:10.1038/s41598-020-63407-8)
Supplement: Supplementary file 1 — Supplementary information [file 41598_2020_63407_MOESM1_ESM.pdf]

Supplementary Data

Comparative quantification of chlorophyll and polyphenol levels in grapevine leaves sampled from different geographical locations.

Elísabet Martín Tornero [1], Ricardo Nuno Mendes de Jorge Páscoa* [2], Anunciación Espinosa Mansilla [1], Isabel Durán Martín-Merás [1], João Almeida Lopes [3]

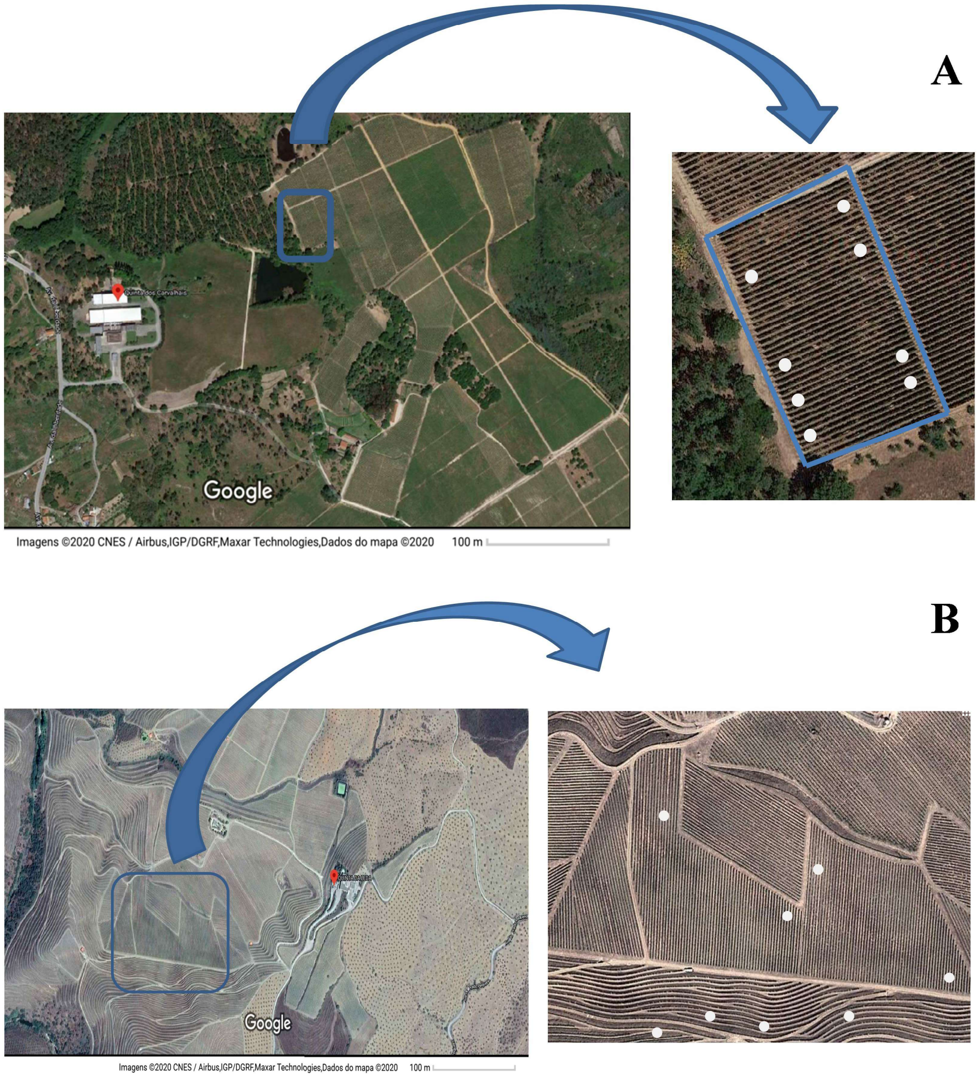

Figure S1. Sampling spots of Quinta dos Carvalhais (A) and Quinta da Leda (B) vineyards.

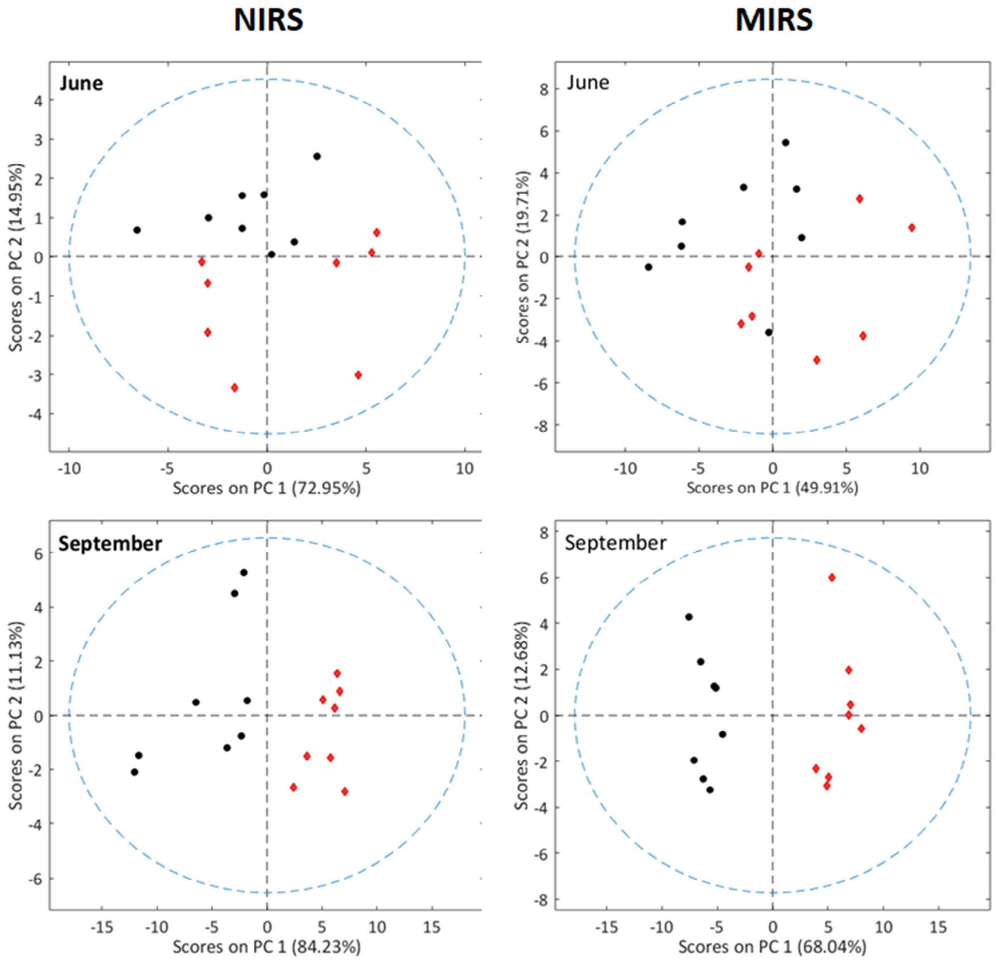

Figure S2. PCA score plot of the two first components obtained for NIR and MIR spectra from samples collected in both geographical regions in June and September considering the entire spectral range. QC and QL samples were marked at black and red, respectively.

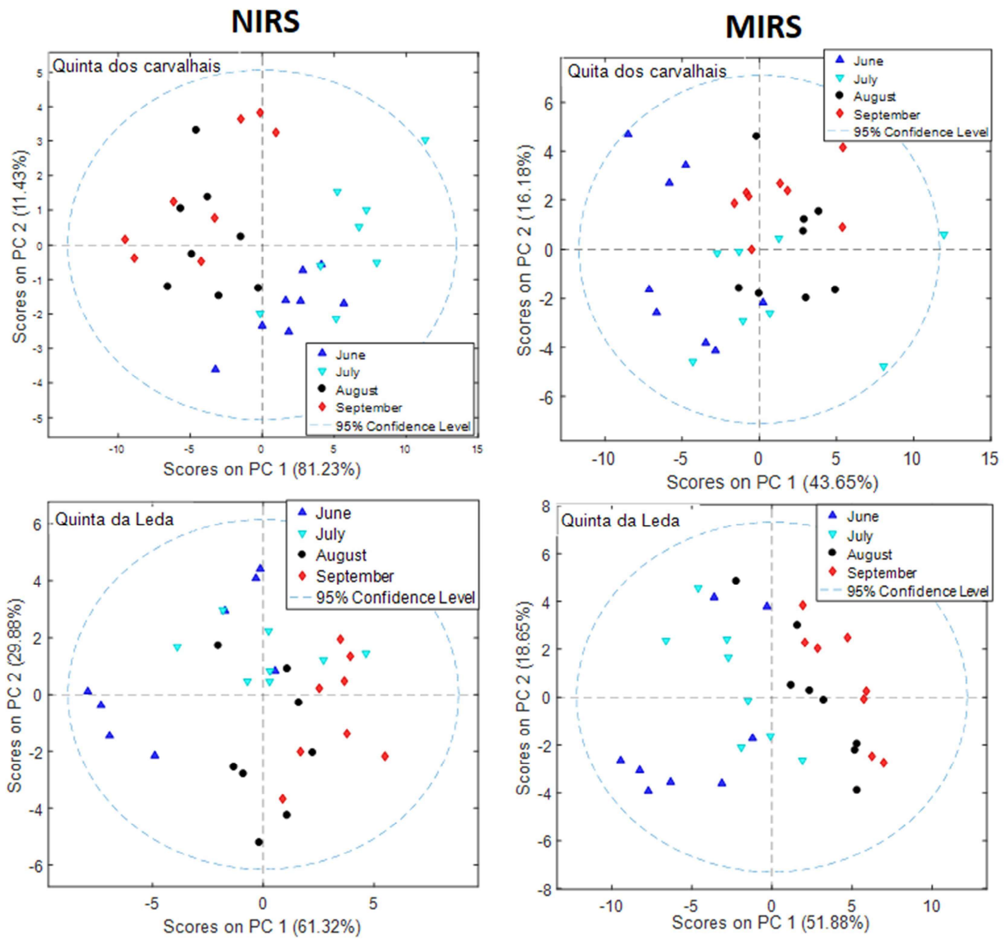

Figure S3. PCA score plot of the two first components obtained for NIR (A) and MIR (B) spectra for both geographical regions considering different sampling dates.
